# Elevated International Normalized Ratio Due to Apixaban in Patient With End-Stage Renal Disease on Hemodialysis

**DOI:** 10.7759/cureus.25907

**Published:** 2022-06-13

**Authors:** Navkirat Kahlon, Sishir Doddi, Ying Ning, Basil Akpunonu, Julie Murphy

**Affiliations:** 1 Hematology and Medical Oncology, College of Medicine and Life Sciences, The University of Toledo, Toledo, USA; 2 Medicine, College of Medicine and Life Sciences, The University of Toledo, Toledo, USA; 3 Oncology, College of Medicine and Life Sciences, The University of Toledo, Toledo, USA; 4 Internal Medicine, College of Medicine and Life Sciences, The University of Toledo, Toledo, USA; 5 Pharmacology, College of Medicine and Life Sciences, The University of Toledo, Toledo, USA

**Keywords:** eliquis, high inr, supratherapeutic, doacs, renal dysfunction, hemodialysis, elevated inr, apixaban

## Abstract

Apixaban is known to prolong international normalized ratio (INR) per some observational and in vitro studies. In patients with elevated INR secondary to apixaban use, median INR of 1.4-1.7 has been reported. Extreme elevation in INR is rare with apixaban. In patients with end-stage renal disease (ESRD) on hemodialysis (HD), there are no labeled indications for apixaban use; however, there are some pharmacokinetic data supporting its use in such patients.

We present a case of a 68-year-old Hispanic man with ESRD who presented to the emergency room (ER) with INR of 27.42. INR testing was done as a part of routine workup in rehabilitation facility. Medication list was reviewed and included apixaban 2.5 mg twice daily which was recently started for postoperative thromboprophylaxis. INR testing was repeated for confirmation in ER and was reported as >18.5 and prothrombin time >200 seconds. His liver function tests were stable as compared to baseline testing five days ago with normal bilirubin, low normal transaminases, and mild hypoalbuminemia. The patient didn’t have any active bleeding. An elevation of INR to >20 with apixaban is a rare event. No other factors including patient characteristics, laboratory results, co-existing conditions, or other medications except the direct oral anticoagulant (DOAC) were found to be responsible for elevated INR. Liver cirrhosis or vitamin K deficiency as cause for INR elevation was ruled out as the baseline INR was normal prior to starting apixaban, liver function tests were stable and INR normalized again shortly after discontinuing the medication. Plasma concentration of DOACs has been found to be correlating with the INR according to a pharmacokinetic study which potentially means that the high INR likely was secondary to high serum concentration of apixaban in this patient. However, INR monitoring is not recommended for monitoring anticoagulant activity of DOACs. As of note, renal clearance accounts for 27% of apixaban clearance.

Pharmacokinetic studies have concluded that half dose apixaban, i.e., 2.5 mg twice daily in patients on hemodialysis (dose used in this case) results in drug exposure similar to that of the standard dose of 5 mg twice daily in patients with preserved renal function. Future studies are necessary to address questions about safety of DOACs in patients with ESRD, further elucidate the clinical significance of such high INR values associated with DOACs, and establish appropriate management guidelines. Andexanet alfa has since been approved for apixaban reversal in patients with life-threatening bleeding; however, would not be indicated in such cases when there is no evidence of bleeding.

## Introduction

Apixaban is a reversible inhibitor of coagulation factor Xa that is indicated for treatment of deep venous thrombosis (DVT) and pulmonary embolism (PE), reducing the risk of recurrent venous thromboembolism (VTE) following initial therapy, reducing risk of stroke and systemic embolism in nonvalvular atrial fibrillation (AF), and postoperative venous thromboprophylaxis following hip or knee replacement surgery [1]. In setting of renal impairment, apixaban has been approved only for atrial fibrillation, to be administered without the need for regular international normalized ratio (INR) monitoring; however, there are no specific indications for patients with end-stage renal disease (ESRD) on hemodialysis (HD) [2]. Clinical trials historically have excluded patients with end-stage renal disease. However, there are some pharmacokinetic data supporting its use in such patients. Renal clearance accounts for 27% of total apixaban clearance. Apixaban can be associated with elevated INR regardless of renal dysfunction; however, the clinical significance of elevated INR is unknown [3]. Maximum concentration of apixaban is reached 1.5-3.3 hours after administration [4]. Kovacevic et al. reported median INR of 1.4-1.7 in inpatient population receiving apixaban [5]. Plasma concentration of direct oral anticoagulants (DOACs) has been found to be correlating with that of the INR [6]. We present a case with extremely high INR which is rare with apixaban use.

This case report was previously presented as a clinical vignette poster in a national internal medicine meeting, i.e., The American College of Physicians Internal Medicine Meeting in Philadelphia, Pennsylvania (held in April 11-13, 2019).

## Case presentation

We report a case of a 68-year-old Hispanic man (59.2 kg, BMI 21.7 kg/m^2^) who was referred to the emergency room (ER) for elevated prothrombin time (PT) and INR. It was done as part of a routine admission testing panel at rehabilitation facility where he was sent for postoperative care after surgical repair and internal fixation of a bimalleolar right ankle fracture. Past medical history includes ESRD on HD, liver cirrhosis, and chronic hepatitis C. Patient denied any obvious bleeding from nose or mouth and blood in urine or stools. He reported no new headaches, focal numbness, or weakness. His medication list included apixaban 2.5 mg twice daily (off-label use) for venous thromboembolic event prophylaxis which he started receiving during recent hospitalization for surgery. It was started as the patient was immobile after surgery and was deemed to be at high risk for VTE. He had received six doses so far and his last dose of apixaban was about 12 hours prior to presentation. He received hemodialysis 36 hours before the presentation. He was not taking any over-the-counter medication, herbal supplements, or any other medication known to cause high INR. The patient was hemodynamically stable. The physical examination was unremarkable except for ecchymotic discolorations at the surgical site. One day prior to the surgery (five days ago), INR was reported to be 1.2. At the rehabilitation center, an INR of 27.42 was recorded and at the ER an INR greater than 18 was confirmed with a PT of >200 seconds. Mixing studies revealed that PT did not fully correct, suggesting possible in vivo or ex vivo circulating inhibitors. Antiphospholipid antibodies were negative. His liver function tests were stable with normal bilirubin, low normal transaminases, and mild hypoalbuminemia.

Per ER protocols, the patient was given 10 mg of IV vitamin K. INR was found to be 2.0, five hours later. The following day, the patient had an INR normalized to 1.1.

## Discussion

INR elevation greater than 20 is rare with apixaban when compared to rivaroxaban [3]. Median INR of 1.4-1.7 in inpatients receiving apixaban has been reported [5]. A review of patients developing hemorrhagic cardiac tamponade in setting of apixaban use showed majority of these patients had elevated INR of 1.3-5.2 and renal dysfunction [7]. An additional review by Shingina et al. concluded that in the context of nonvariceal upper gastrointestinal bleeding, an elevated INR does not predict rebleeding; however, an elevated value defined as above 1.5 is associated with increased patient mortality [8].

According to the package insert of Eliquis (apixaban) INR is not indicated to monitor the toxicity or efficacy of the drug. Therefore, in the absence of major bleeding, monitoring for signs or symptoms of severe bleeding is all that is indicated [9]. In a case like this, administration of vitamin K does not have a beneficial effect as vitamin K is not expected to neutralize the effect of apixaban. Liver cirrhosis or vitamin K deficiency (where vitamin K can be useful) as cause for INR elevation in our case was ruled out as the baseline INR was normal prior to starting apixaban, liver function tests were stable and it normalized again shortly after discontinuing the medication. In the case of uncontrolled or life-threatening bleeding due to apixaban, andexanet alfa can be used as reversal agent [10].

Eliquis package insert states that its efficacy and safety studies did not enroll patients with ESRD on HD [9]. There is little data supporting the use of apixaban in patients with ESRD on hemodialysis in the form of pharmacokinetic studies. Plasma concentration of direct oral anticoagulants (DOACs) has been found to be correlated with that of the INR which means that the elevated INR in our case could have been secondary to supratherapeutic levels of apixaban [6]. Mavrakanas et al. looked at pharmacokinetics of apixaban at steady state in hemodialysis patients and concluded that half dose apixaban, i.e., 2.5 mg twice daily in patients on hemodialysis (dose used in this case report) resulted in drug exposure similar to that of the standard dose of 5 mg twice daily in patients with preserved renal function and was concluded to be a reasonable choice instead of warfarin for stroke prevention in patients on dialysis [11]. However, 5 mg twice daily dose in such patients resulted in supratherapeutic levels [11]. Wang et al. also found that the pharmacokinetics of apixaban does not significantly differ between patients with ESRD and those with preserved renal function [1]. These studies supporting use of half dose apixaban in hemodialysis patients have led to increased use in these patients given the ease of use without INR monitoring. However, elevated INR level like in our case raises questions about safety of its use.

In ESRD patients, dialysis does not affect the apixaban drug levels [11]. Thus, apixaban levels can’t be adequately brought down with urgent HD if an emergency situation like life-threatening bleeding arises due to supratherapeutic drug levels. Caution should be executed prior to making the decision to use a DOAC in patients with abnormal renal function, particularly ESRD patients and randomized clinical trials should be done to answer questions about the safety of these agents in such patients.

## Conclusions

Even though the pharmacokinetic data supports the use of half dose (2.5 mg twice daily) apixaban in patients with renal dysfunction including ESRD patients, unpredictable supratherapeutic levels can possibly be seen in rare cases. This can potentially result in bleeding complications. Routine testing and monitoring of apixaban with PT/INR is not recommended. However, routine standard testing panels used these days at various institutes can lead to INR testing in a patient on apixaban and result in an incidental finding of high INR. Further studies are necessary to elucidate the significance of such high INR values with direct oral anticoagulants such as apixaban, especially in patients with impaired renal function. Standard guidelines for the management of elevated INR in such patients can be established only after the clinical relevance is established. Per the data available so far, observation for signs or symptoms of severe bleeding is likely all that is needed unless patient has a severe or life-threatening bleed which would warrant use of reversal agent andexanet alfa. Until adequate safety data on apixaban in hemodialysis patients is available, its use in patients with ESRD should be avoided. If it is used in such patients, close observation for signs and symptoms of bleeding is warranted.
